# The Relationship Between Structural Game Characteristics and Gambling Behavior: A Population-Level Study

**DOI:** 10.1007/s10899-014-9477-y

**Published:** 2014-06-13

**Authors:** Tony Leino, Torbjørn Torsheim, Alex Blaszczynski, Mark Griffiths, Rune Mentzoni, Ståle Pallesen, Helge Molde

**Affiliations:** 1 Department of Clinical Psychology, University of Bergen, Christiesgate 12, 5015 Bergen, Norway; 2 Department of Psychosocial Science, University of Bergen, Christiesgate 12, 5015 Bergen, Norway; 3 School of Psychology, The University of Sydney, Griffith Taylor - A19, Sydney, NSW 2006 Australia; 4 International Gaming Research Unit, Psychology Division, Nottingham Trent University, Burton Street, Nottingham, NG1 4BU UK

**Keywords:** Structural characteristics, Reward characteristics, Bet characteristics, Video lottery terminal, Multilevel modelling, Behavioral tracking data

## Abstract

The aim of this study was to examine the relationship between the structural characteristics and gambling behavior among video lottery terminal (VLT) gamblers. The study was ecological valid, because the data consisted of actual gambling behavior registered in the participants natural gambling environment without intrusion by researchers. Online behavioral tracking data from *Multix,* an eight game video lottery terminal, were supplied by *Norsk*-*Tipping* (the state owned gambling company in Norway). The sample comprised the entire population of *Multix* gamblers (N = 31,109) who had gambled in January 2010. The individual number of bets made across games was defined as the dependent variable, reward characteristics of a game (i.e., payback percentage, hit frequency, size of winnings and size of jackpot) and bet characteristics of a game (i.e., range of betting options and availability of advanced betting options) served as the independent variables. Control variables were age and gender. Two separate cross-classified multilevel random intercepts models were used to analyze the relationship between bets made, reward characteristics and bet characteristics, where the number of bets was nested within both individuals and within games. The results show that the number of bets is positively associated with payback percentage, hit frequency, being female and age, and negatively associated with size of wins and range of available betting options. In summary, the results show that the reward characteristics and betting options explained 27 % and 15 % of the variance in the number of bets made, respectively. It is concluded that structural game characteristics affect gambling behavior. Implications of responsible gambling are discussed.

## Introduction

Gambling machines [e.g., pokies, slot machines and video lottery terminals (VLTs)] incorporates a large number of structural characteristics. These machines have evolved from a simple three wheeled mechanical devices into multigame, digitalized and electromagnetic machines with advanced audiovisual effects and touch-screens with numerous gaming features and structural modifications (Griffiths 1993; McCormack and Griffiths 2013; Parke and Griffiths 2006; Schüll 2012). Structural characteristics refer to the distinct features and properties of a gambling apparatus or a game (Griffiths 1993). Primary structural characteristics include the core technology of a game, such as reinforcement schedules and betting configurations, whereas secondary characteristics include artwork, buttons, lights and sound effects (Livingstone and Woolley 2008, for more comprehensive reviews see Griffiths 1993; King et al. 2010; Parke and Griffiths 2006, 2007; Wood et al. 2004).

Game preference and gambling behavior appear to be associated with individual factors (e.g., traits and motives), and situational factors (e.g., availability, accessibility and diversity of offered games) (Smith et al. 2007). However, both theoretical assumptions and empirical findings suggest that the structural characteristics of a game influence gambling behavior. By satisfying individual needs and reinforcing gambling behavior, it has been suggested that structural characteristics may lead to the acquisition, development and sustainment of gambling behaviors (Griffiths 1993). Among others, a primary motivation to gamble has been linked to game features such as the chance of winning and hitting the jackpot (Binde 2012). In line with learning theory, gambling behavior may become conditioned to certain structural game characteristics through operant and classical conditioning (Blaszczynski and Nower 2002). These processes may shape and maintain gambling behavior over time. For example, structural game characteristics, such as event frequency, payout interval and reward distribution have often been associated with the development of gambling behaviors that are hard to extinct (Dowling et al. 2005).

Both laboratory studies and self-reports have examined the relationship between the reward characteristics of a game and gambling behaviors (e.g., Coates and Blaszczynski 2012; Dixon et al. 2006; Haw 2007; Livingstone and Woolley 2008; McCormack and Griffiths 2013; Weatherly and Brandt 2004). Livingstone and Woolley (2008) examined the attractiveness of several gaming machine features based on a telephone survey of 180 Australian regular gamblers. Their results indicated that reward characteristics (size and frequency of wins) and free games (such as free spins) were the most attractive structural characteristics of a game. Although self-reports have shown a general preference for reward characteristics, experimental studies of the relationship between gambling behavior and reward characteristics has provided less conclusive results. Some studies have failed to support an association between gambling behavior and reward characteristics (Weatherly and Brandt 2004); others have found only partial support for a relationship (Haw 2007), whereas others have reported support for a relationship between gambling behavior and reward characteristics (e.g., Coates and Blaszczynski 2012; Dixon et al. 2006). Dixon et al. (2006) found that gamblers prefer games with more frequent but smaller wins to games with less frequent and big wins. In line with these findings, Coates and Blaszczynski (2012) found that individuals prefer games with more frequent wins and higher payback percentages. These findings suggest that players will prefer games with more frequent wins and higher payback percentages when they are able to discriminate and choose between games with different reward characteristics. Findings suggest that in-session gambling behaviors are sensitive to the credit value of a win. In-session gambling behaviors appear to slow down after larger wins but continues unchanged by smaller wins (Delfabbro and Winefield 1999). Overall, these findings suggest that reward characteristics influence gamblers; in particularly, gamblers appear to prefer games with more frequent small wins and/or a higher payback percentage. Furthermore, games with a smaller average win size appear to be associated with a higher degree of gambling responses, such as bets made.

The betting opportunities allowed in a game also appear to influence gambling behavior. Research findings indicate that the maximum size of a bet allowed in a game is positively associated with time spent, number of bets made and monetary losses in a gambling session (Sharpe et al. 2005). Multiplier potential refers to the opportunity to vary odds and stakes in a game (Griffiths 1993). Individuals appear to be motivated to vary the odds and stakes of a bet. For example, Livingstone and Woolley (2008) found that the majority of participants (86 %) bet on multiple or maximum pay lines independent of bet size, whereas approximately half (52 %) of the participants had a strategy that involved a minimum bet on multiple lines or the maximum number of pay lines. The attractiveness of this feature seems to be related to maximizing the frequency of wins. Findings indicate that the number of pay lines, but not bet multiplication, is a predictor of average bet size (Haw 2009). Haw (2009) suggests that this feature establishes a contingency between the feature itself and game play that reinforces gambling behavior. Hence, it can be assumed that the size of a bet will be higher than the cost to play a game, and games that offer more advanced betting opportunities, such as changing the odds, will be associated with more bets.

Taken as a whole, empirical findings in the literature indicate that game preference and gambling behaviors are related to the structural characteristics of a game. Gambling behavior may therefore be viewed as a function of the individual factors, situational factors and structural game characteristics that determines participation in the activity (McCormack and Griffiths 2013). However, no previous study has examined the relationship between structural characteristics of games’ and population-level gambling behavior. Furthermore, findings from the laboratory and self-report studies should be examined and replicated within a natural setting (Peller et al. 2008; Shaffer et al. 2010). In line with recent technological developments, online behavior tracking provide a unique opportunity to examine gambling behavior in the natural setting of the gambler without intrusion by researchers (Delfabbro et al. 2012; Shaffer et al. 2010). This method is already used in Internet gambling research (see reviews by Delfabbro et al. 2012; Griffiths and Whitty 2010; Shaffer et al. 2010), however due to network based solutions in offline gambling machines this method is also emerging among land based gambling terminals, providing a unique opportunity to examine actual gambling behavior in an ecologically valid setting.

The Norwegian gambling market was regulated in 2007, and privately operated gambling machines were replaced with newer video lottery terminals (VLTs) run by the state owned *Norsk Tipping*. *Multix* was introduced in 2008 (Norsk-Tipping 2010) and is available in public places, such as convenience stores. *Multix* is a multi-game VLT, designed with two 22 inch screens (1,680 × 1,050 resolution), a sound system with a subwoofer, three buttons, a smart card reader and a pin pad. It is a cashless system that can only be accessed using a personal smart card that is linked to the players’ account through their Norwegian social security number. Furthermore, the smart card enables the system to monitor, track and register an individual’s gambling behavior and gambling history. Tracking data of actual gaming behavior offers a unique opportunity to examine the relationship between the structural characteristics of gambling machines and gambling behavior.

The aim of this study is to examine the relationship between structural characteristics and gambling behavior in an ecologically valid setting using actual gambling behavior registered in the gamblers’ natural environment. According to behavioral theory and previous empirical findings, we expected that the structural characteristics would be associated with gambling behavior. More specifically, we expected that gambling behavior would be positively associated with payback percentage, frequency of wins, size of jackpot and the opportunity to vary the odds and size of a bet in a game. Furthermore, in line with findings by Delfabbro and Winefield (1999), we expected that the number of bets made would be negatively related to the average size of wins.

### Multix as a Responsible Gambling Device

Although new gambling technology is often a source of public concern (Griffiths 1999; Peller et al. 2008), technological inventions may also enhance responsible gambling and player control by modifying structural characteristics and gambling features (Blaszczynski et al. 2013, 2004; Peller et al. 2008). Electronic gambling machines afford a greater opportunity to control for structural and environmental factors that influence game play (Wood et al. 2004). As such, preventive gambling features can be differentiated between supply and demand reductions (Cantinotti and Ladouceur 2008).

Demand reduction aims to moderate player behavior by informing the gambler of their current gambling status (Cantinotti and Ladouceur 2008). *Multix* displays credit the balance and total amount of money won in NOK which might help the gambler keep better track of the money they have spent. In addition to a physical help button, all games have an interactive help button that presents information about gambling problems. Diagnostic self—tests, self-limiting and self-exclusion options are available in *Multix*. Self-tests can help players to determine if their gambling activity indicates gambling problems. The gambler also has the opportunity to set voluntary pre-commitment limits of use in addition to partial and full exclusion in *Multix*. Self-exclusion cannot be reversed for minimum of 100 days. Empirical research using behavioral tracking data has demonstrated the effectiveness of setting both time and money limits to protect players that have the highest gaming intensity (Auer and Griffiths 2013).

Supply reduction refers to alterations in electronic game machines (EGMs) that restrict the use (Cantinotti and Ladouceur 2008). *Multix* players must be 18 years old and the terminals cannot be accessed between midnight and 07:00 a.m. However, it should be noted that the system also aims to regulate gambling by setting mandatory time and expenditure limits. After 60 min of continuous gambling the player is excluded from all *Multix* terminals for 10 min. In 2010 gambler’s individual monetary losses could not exceed 400 NOK within 24 h and 2200 NOK within a month (In 2013 these limits were 600 NOK/day and 2,500 NOK/month). If these limits were exceeded, the gambler was excluded from gambling for the remainder of that day/month. These behavioral regulations are enforced electronically by a personal smart card that enables the system to monitor, track and register an individual player’s behavior. However, the question remains whether these gambling features have had the intended effect on problem gambling in Norway.

## Method

Data were supplied by *Norsk*-*Tipping*, the state owned gambling company in Norway and consist of individual gambling behaviors (number of days, sessions, wagers and expenditure) recorded in January 2010. The data were collected unobtrusively through online behavioral tracking.

### Sample

The sample consisted of all individuals who gambled on a *Multix* terminal in January 2010. The sample included 31,109 players, of which 7,110 (22.6 %) were women and 24,299 (77.4 %) were men. Ages ranged from 18 to 102 years old with a mean of 43.1 [standard deviation (SD) 15.9] years old. The mean age was higher among women (m = 47.3 years old, SD = 16.7) than among men (m = 41.8 years old, SD = 15.5).

Eight *Multix* games were available in January 2010. Five games could be characterized as games of pure chance [*Jokerdryss Bling Bling*, *Ballpower*, *Arishinko* (Pachinko), *Swing* (Wheel of Fortune) and *Roulette*], and three games could be characterized as semi skill games [*Black Jack*, *Opp og Ned* (card games) and *Knipsekassa*] (Turner 2008).

### Measures and Gambling Indicators

The gambling indicator used was the total number of bets an individual made in a game.

The only available demographic variables obtained were age and gender.

### Game Characteristics

The fixed reward characteristics of a game across gamblers were the payback percentage (return to player; RTP), average hit frequency, size of win, size of jackpot and availability of bonus features within a game. More specifically, the RTP represents the ratio between the aggregate sum of wins across all players divided by the aggregated sum of the bets made by all players within a game. A higher RTP represents a lower expected credit loss per bet.

The average hit frequency of a game represents the ratio between the aggregate number of bets made divided by the aggregated number of wins obtained by all players within a game. A lower quotient represents more frequent wins per bet in a game (i.e., fewer bets needed to obtain a win of any size). However, although multiple wins per bet could be obtained in some of the games (*Jokerdryss Bling Bling*, *Ballpower* and *Black Jack*), this measure recorded one (1) win per bet regardless of the number of wins within the same wager (Norsk-Tipping, personal communication, September 12, 2013). Hence, the absolute hit frequency in some games might actually have been lower than the estimate suggests.

The average size of a win in a game represents the ratio between the sum of all wins divided by the number of aggregate number wins across all players. A higher number reflects a higher average win size within a game.

Jackpot size represents the maximum win within a game. Four of the games (*Jokerdryss Bling Bling*, *Ballpower*, *Arishinko*, *Swing*) had a maximum jackpot of 1,500 NOK; *Roulette* had a maximum jackpot of 1,440 NOK; *Knipsekassa* had a maximum jackpot of 500 NOK; *Opp og Ned* had a maximum jackpot of 400 NOK; and *Black Jack* had a maximum jackpot of 125 NOK.

A dummy variable indicated the presence (1) or absence (0) of bonus game feature. The games that included a bonus feature were *Arishinko*, *Ballpower*, *Joker Dryss Bling Bling* and *Knipsekassa*.

To measure the range of the bet size allowed within a game, the difference between the maximum and minimum bet sizes were calculated (i.e., the range was the maximum minus the minimum stakes in a game). Games with a smaller range of allowed bet size have a smaller variation, whereas games with greater range have more variation in allowed bet size.

All of the games included the opportunity to vary the stakes of a bet. Furthermore, games with more advanced betting options were (with advanced betting option in parenthesis) *Arishinko* (bonus pockets; more advanced price structures and bonus games), *Ballpower* (multiple bet lines), *Black Jack* (multiple simultaneous games), *Swing* (choose winning symbols with different odds to win) and *Roulette* (bet on multiple numbers). A dummy variable “advanced betting options” represented games with betting options beyond stake multiplication.

### Procedure and Ethical Considerations

A multilevel framework was used to analyze the data. Multilevel models should be conducted when observations are not independent, but are clustered/nested within contextual factors (Hox 2010). Treating observations as independent when they are not, may threaten the validity of a model (Bickel 2007). However, because gambling behaviors may be nested within both individuals and games, a cross-classified multilevel random intercepts model was defined to estimate the relationship between structural game characteristics and gambling behavior. The model defined gambling behavior (i.e., individual bets made in a game) as a function of (1) structural game characteristics and (2) demographic variables nested within games and individuals at the same level.

$$\begin{aligned} {\text{Bets}}\,{\text{made}}_{{{\text{i}}\left( {\text{jk}} \right)}} & = & & & \gamma_{00i(jk)} + \gamma_{01} RTP_{(j)} + \gamma_{02} Frequency\,of\,wins_{(j)} + \gamma_{03} Size\,of\,wins_{(j)} + \gamma_{04} Jackpot_{(j)} \\ & & & \quad + & \gamma_{05} Bonus_{(j)} + \gamma_{06} Range\,Bet_{(j)} + \gamma_{07} Advance\,bet\,options_{(j)} + \gamma_{8} Female_{\left( k \right)} \\ & & & \quad + \gamma_{9} Age_{\left( k \right)} + u_{0j} + v_{0k} + e_{{i\left( {jk} \right)}} \\ \end{aligned}$$

The model suggests that the number of bets made is a function of the aggregate game characteristics and demographic variables where the intercepts are allowed to vary between games (j) and individuals (k). The fixed effects of the model provides mean estimates of the explanatory variables, whereas the random effects provide variations in the intercepts among individuals and games.

Prior to the analysis, the data were screened for errors. In a total of 4,690 cases, a sum of wins was recorded but no frequency of wins. This lack of data can occur when a gambling terminal is forced offline (due to e.g., power shortage or communication problems with the server) (Norsk-Tipping, personal communication, July 2, 2013). The server registers the outcome of the wager and displays it to the player at the next log in. For these cases, the outcome was determined during an earlier period, but registered in January. To overcome this issue, the sum of wins was set to zero if no frequency of wins was observed.

Data from three subjects were deleted. The first case was a subject with a total personal loss of 2,900 NOK, exceeding the *Multix* limit of 2,200 NOK. *Norsk*-*Tipping*’s database for checking individual losses was temporary offline during this period (Norsk-Tipping, personal communication, December 2, 2013). Similarly, two subjects had an average bet size of 55 and 60 NOK in *Black Jack*, exceeding the *Multix* limit of 50 NOK, which is attributed to communication problems with the server. These exclusions left 31,406 individuals from the original sample.

The distribution of bets made was checked statistically and with plots. Bets made had a strong positive skew (4.9) and a positive kurtosis (41.6), indicating departure from normality. Several transformations were examined before a logarithmic transformation was ultimately chosen. Although the logarithmic transformation improved the statistical properties of the distribution in bets made (skew: 0.70, kurtosis: 2.2), histograms still revealed deviations from normality. However, because the statistical properties of the dependent variable were satisfactory and deviations from normality are common within large samples, the data appear to be suitable for parametric analyses.

The analysis of the cross-classified multilevel model followed a “bottom—up” approach suggested by Hox (2010). First, an *intercept*-*only model* (null model – Model 1) was investigated. Intra-class correlation coefficients (ICC) were calculated, in addition to estimating the deviance of the model. Second, the structural characteristics of games were included as fixed factors to examine their effects (Model 2). Third, to estimate the effects of the structural characteristics according to control variables, demographic variables were included as fixed factors in the final model (Model 3). Finally, a standardized model, Z-model, was analyzed to examine the relative contribution of the structural characteristics and the demographic variables. The standardization of the game characteristics and the demographic characteristics were performed in separate data files and contained only the aggregate game characteristics and demographic characteristics, respectively. These values were then merged into the main data file.

Ethical approval for the study was granted by the Norwegian Social Science Data Services (NSD). Individual consent to use individual gambling data anonymously is covered in the contract between the individual and *Norsk*-*Tipping*. To ensure that the participants remained anonymous, *Norsk*-*Tipping* created new identity codes for each participant and the key to reverse the process was deleted prior to data export.

The data were analyzed in STATA 12 (StataCorp 2011).

## Results

### Sample Characteristics

Descriptive statistics show that women (M_log_ = 4.50, SD_log_ = 2.31) placed more bets than men (M_log_ = 4.06, SD_log_ = 2.26), t(76,328) = −22.43, *p* < 0.001. However, the magnitude of the differences was very small (eta^2^ = 0.007). A main effect of age and (log)bets made was found, F(3,76326) = 2,229.14, *p* < 0.001, showing that bets increased with age. The magnitude of the differences in the means between different age groups was moderate (eta^2^ = 0.08).

### Gambling Participation

Most individuals gambled on several games in January. Of the total sample 11,083 (35.3 %) bet on only one game, 8,106 (25.8 %) bet on two games, 5,716 (18.2 %) bet on three games, 3,242 (10.3 %) bet on four games, 1,640 (5.2 %) bet on five games, 892 (2.8 %) bet on six games, 462 (1.5 %) bet on seven games, and 268 (0.8 %) bet on all eight games. These results show that 59. 5 % gambled on two to four games, and 5.1 % gambled on five or more games.

Table 1 show the participation across games. A total of 76,330 games were played in January, and each individual played an average of 2.4 games in January. Participation was higher in some games than others. Pure chance games were more popular than the semi-skill or casino games. The greatest number of bets and the highest expenditures (median) were made in *Ballpower*, followed *by Jokerdryss Bling Bling* and *Arishinko*, whereas the fewest numbers of bets and the lowest expenditure were placed in *Opp og Ned*. The relative positions of the other games varied with respect to the number of days gambled and bets made. It should also be noted that *Jokerdryss Bling Bling* had the highest participation rate, which may indicate that most players occasionally gambled on this game.

**Table 1:** Descriptive statistics of individuals’ gambling behaviors of games in *Multix* in January of 2010

Game	Participated		Days		No. of bets made		Expenditure in NOK		Bet in NOK	
	N	(%)	m (SD)	Md	m (SD)	Md	m (SD)	Md	m (SD)	Md
JD Bling	20,089	64.58	4.81 (4.59)	3	860.03 (1,503.95)	253	5,739.35 (9,875.45)	1,632	6.77 (2.75)	7.12
Ballpower	15,185	48.82	5.76 (5.35)	4	1,253.28 (1,946.87)	407	5,356.41 (7,975.09)	1,771.5	4.06 (1.35)	4.78
Arishinko	10,937	35.16	3.35 (3.79)	2	336.5 (735.37)	76	2,399.21 (5,244.58)	496	6.44 (2.90)	6.11
Swing	8,385	26.96	2.4 (2.89)	1	46.39 (147.57)	10	836.43 (2,857.62)	100	13.08 (9.72)	10
BlackJack	7,791	25.05	1.99 (2)	1	39.05 (95.49)	13	799.72 (2,374.06)	159	16.59 (13.43)	12.27
Knipsekassa	5,195	16.70	1.78 (1.85)	1	64.73 (214.65)	19	350.69 (1,566.22)	45	3.58 (3.14)	1.83
Opp og Ned	5,162	16.59	1.70 (1.91)	1	25.37 (132.01)	6	249.71 (1,294.74)	30	7.01 (6.25)	5
Roulette	3,856	12.40	1.84 (2.04)	1	86.29 (291.50)	19	699.28 (1,879.36)	120	7.17 (4.94)	5.79

*JD Bling* Jokderdryss Bling Bling. Total N = 76,330. Participated % = Participation n/31,106. Average 1 US$ in January 2010 = 5.73 NOK

All the measurements are rounded to the third decimal

### Structural Game Characteristics Between Games

Table 2 presents the descriptive statistics of structural characteristics of the available *Multix* games. The results show that all of the average bet-sizes were larger than the minimum stakes across all of the games, suggesting that multiplier potential features were common. Furthermore, (1) the minimum stake with the maximum lines in *Ballpower* was 1.25 NOK (25*0.05 NOK), (2) the minimum stake with the maximum bonus pockets in *Arishinko* was 5 NOK (5*1 NOK), (3) the minimum stake in *Black Jack* with multiple games was 3 NOK (3*1 NOK) and the (4) the minimum stake in *Swing* with three winnings symbols was 3 NOK (3*1 NOK). Because the average bet-sizes were higher than the minimum stakes for all of the extra features, it is likely that the gamblers combined multiple winning lines/bonus pockets/multiple games with the bet multiplication feature.

**Table 2:** Descriptive statistics of the structural characteristics between games in *Multix* in January of 2010

Game	Bet characteristics			Reinforcement characteristics					Time characteristics	
	Min–max bet	Bet	Bet options	Min–max win	RTP (%)	Hit freq.	Size of win (NOK)	Bonus	Min duraton (s)	Actual duration (s)
JD Bling	2–10 NOK	6.67 NOK	No	0.2–1500 NOK	91.86	7.68	47.08 NOK	Yes	3	4.5
Ballpower	0.05–5 NOK	4.27 NOK	Reelpower	0.25–1500 NOK	89.63	8.47	32.45 NOK	Yes	3	7
Arishinko	0.1–10 NOK	7.13 NOK	Bonusgames	0.2–1500 NOK	93.04	6.71	44.56 NOK	Yes	3	6
Swing	1–30 NOK	18.03 NOK	Win symbols	2–1500 NOK	90.52	6.71	109.45 NOK	No	10	10
Black Jack	1–50 NOK	20.48 NOK	Multiple games	1–125 NOK	90.34	2.46	45.49 NOK	No	3	15
Knipsekassa	1–10 NOK	5.42 NOK	No	3–500 NOK	89.50	5.91	28.68 NOK	Yes	3	3.5
Opp og Ned	1–15 NOK	9.84 NOK	No	0.5–400 NOK	84.56	6.43	53.48 NOK	No	3	7
Roulette	1–40 NOK	8.10 NOK	Win number	2–1440 NOK	84.88	4.33	29.81 NOK	No	13	13

*JD Bling* Jokerdryss Bling Bling. Average 1 US$ in January 2010 = 5.73 NOK. Individual within game behavior aggregated to game level

Bet = sum expenditure/# of wagers. RTP = sum of wins/sum of expenditures. Hit frequency = # of bets made/# of wins. Size of win = sum of wins/# of wagers. All the measurements are rounded to the third decimal

Reward characteristics differed between games. Five of the games offered a jackpot over 1,000 NOK, whereas three of the games offered a jackpot equal to or less than 500 NOK. Pure chance games and the casino games were associated with higher maximum wins, whereas the semi-skill games were associated with lower maximum wins (wins of 500 NOK or less).

The fixed average payback percentage (i.e., RTP) of a game across all gamblers ranged from 84.56 to 93.04 % (m = 89.29, SD = 3.05) and was positively related to participation. *Opp og Ned* and *Roulette* had a lower RTPs, whereas *Arishinko* and *Jokerdryss Bling Bling* had a higher RTPs.

The fixed average hit frequency of a game across all gamblers ranged from 2.46 to 8.47 (m = 6.09, SD = 1.90). Notably, the most popular games had less frequent payouts on average, whereas *Black Jack* had the most frequent payouts.

The fixed average size of a win of a game across all gamblers ranged from 28.68 NOK to 109.45 NOK (m = 48.87, SD = 26.09). The average win size in *Swing* deviated from other games: Z [(109.45-48.87)/26.09] = 2.32, indicating that *Swing* had a significantly higher average payout size than the other games.

Four of the games (*Arishinko*, *Ballpower*, *Jokerdryss Bling Bling* and *Knipsekassa*) had bonus features. Except for *Knipsekassa,* games with bonus feature were associated with a higher bet frequency.

All of the games, except *Swing* and *Roulette*, had a minimum theoretical duration of 3 s. However, there was a discrepancy between the minimum duration of a game and the actual duration of a game. Most games, except for *Swing* and *Roulette,* had a longer actual duration. This difference may be associated with the type of game. In some games, longer duration may be associated with occurrence of bonus features that lengthens the sequence, whereas in other games, such as semi-skill games, the actual time may be positively associated with best betting strategy.

### Bivariate Analysis of Structural Characteristics and Gambling Indicators

Table 3 presents bivariate relationships between number of bets made and structural game characteristics. Spearman’s rho was chosen to estimate the strength between structural characteristics and bets made. The relationship between bets made and game characteristics ranged from small to strong. The analysis show that all of the game characteristics were positively related to bets made, except for size of wins and range of bet. The findings suggest that the game characteristics of bonus features, jackpot size and RTP, less frequent wins and smaller wins were positively associated with more bets made. Furthermore, games with a small difference in maximum and minimum bet size and the opportunity to place additional bets on lines/bonus features/simultaneous games were associated with more bets made. However, the association with advanced betting options was weak, indicating no overall effect.

**Table 3:** Spearman’s correlation of bets made and structural game characteristics

	2	3	4	5	6	7	8
1. Bets made	0.232	0.543	−0.163	0.52	0.433	−0.529	0.08
2. RTP	–						
3. Hit frequency	0.194	–					
4. Size of win	0.326	−0.132	–				
5. Bonus	0.401	0.735	−0.41	–			
6. Jackpot size	0.581	0.795	0.131	0.613	–		
7. Bet range	−0.024	−0.939	0.314	−0.826	−0.626	–	
8. Advanced betting options	0.027	0.045	−0.307	0.273	0.065	−0.222	–

Bets made = log(bets). N = 76,330. All of the estimates are significant on *p* < 0.001. Bonus games and advanced betting options are dummy variables

The analysis also shows a complex relationship between several of the structural variables. The relationships between hit frequency, jackpot size, bonus features, and range of betting options were strongly associated, indicating multicollinearity. To further examine the relationships between the structural variables, several regression models were conducted to examine the degree variance explained by using predictor variables as both endogenous and exogenous variables. The analysis showed that the other reward characteristics explained a large amount of the variance in bonus games (R^2^ = 0.97). Furthermore, hit frequency and size of wins explained a large portion of the variance in the range of size (R^2^ = 0.90).

Separate regression models for reward characteristics and bet characteristics were conducted. The regression models for bet characteristics (variation in bet sizes and line features in a game) supported the findings of the correlation analysis showing no multicollinearity between the variables. The regression model for the reward characteristics (RTP, hit frequency, size of wins, jackpot size and opportunity for bonus games) showed multicollinearity for bonus games. Models with and without bonus game were investigated. The analysis indicated a change in R^2^ = 0.019 (R_Model with bonus_^2^ = 0.336, R_Model without bonus_^2^ = 0.317). Because the other reward characteristics explained a large amount of the total variance of bonus games, the variable was dropped from the analysis.

### Cross-Classified Multilevel Model of the Relationship Between Structural Characteristics and Betting Behavior

Two cross-classified multilevel models were investigated to examine the relationships among reward, bet characteristics and gambling behavior. Table 4 presents the cross-classified model for the number of bets made and the reward characteristics nested within individuals and games. The null model shows that log(3.59) bets were made on average adjusted for games and individuals. The ICC shows that 42.4 % of the number of bets made can be attributed to contextual factors. Of the total ICC, 31.1 and 11.2 % of the bets made can be attributed to games and individuals, respectively. The high ICC supports the inclusion of games and individuals as random components in the model.

**Table 4:** A cross-classified multilevel model of the relationship between reward characteristics, demographic variables and the number of bets made

Parameters	Model 0	Model 1	Model 2	Z Model
*Fixed effects*				
Intercept	3.59 (0.43)	4.05 (0.17)	4.07 (0.17)	3.63 (0.15)
*Structural characteristics*				
RTP	–	18.74 (5.79)	18.83 (5.74)	0.57 (0.18)
Hit frequency	–	0.25 (0.11)	0.24 (0.11)	0.46 (0.21)
Size of win	–	−0.03 (0.01)	−0.03 (0.01)	−0.73 (0.17)
Jackpot	–	0.0008 (0.0004)	0.0006 (0.0005)^NS^	0.39 (0.21)^NS^
*Demographic characteristics*				
Female	–	–	0.08 (0.02)	0.03 (0.01)
Age	–	–	0.02 (0.00)	0.38 (0.01)
*Random effects*				
$\sigma_{0i}^{2}$	2.79 (0.02)	2.79 (0.02)	2.77 (0.02)	2.77 (0.02)
$\sigma_{0j(Game)}^{2}$	1.51 (0.75)^NS^	0.19 (0.1)^NS^	0.19 (0.1)^NS^	0.19 (0.1)^NS^
$\sigma_{0k(Individual)}^{2}$	0.54 (0.01)	0.54 (0.01)	0.43 (0.01)	0.43 (0.01)
*Model summary*				
−2LL	306,595.26	306,578.85	304,023.96	304,023.96
AIC	306,603.26	306,594.85	304,043.96	304,043.96
R^2^	–	27.1 %	30.0 %	
# of estimated parameters	4	8	10	10

Standard errors are listed in parentheses. Explanatory variables were centered in all of the models, except for the Z-model, in which the scores were standardized prior the analysis. Bets = log(bets)

*NS* Not significant

Model 1 shows that all of the reward game characteristics were positively associated with the number of bets made, except for the size of win, which was negatively related to number of bets made. Interestingly, hit frequency is positively related to the number of bets made, showing that less frequent wins per bet was associated with a higher number of bets. The reward characteristics explained 27.1 % of the total variance in gambling behavior. The inclusion of reward characteristics reduced the random component of games by 87.4 % and had a minimal effect on the other variance components. This result shows that reward characteristics constitute real contextual factors, and a large portion of the variance in number of bets made between games can be attributed to differences in reward characteristics. The inclusion of structural characteristics significantly improved the model (*χ* ^2^ = 16.41, *df* = 4, *p* < 0.01).

Model 2 shows that reward characteristics and demographic variables were related to the number of bets made, except for jackpot size. The final model predicts that higher RTP, less frequent wins, smaller size of average wins, being female and increasing age are positively associated with the number of bets made. The inclusion of demographic variables reduced the individual variance component by 20.4 %, showing that one-fifth of the between individual variance can be attributed to demographic factors. The final model explains 30 % of the total variance in the number of bets made. The inclusion of the demographic characteristics significantly improved the model (*χ* ^2^ = 2,554.96, *df* = 6, *p* < 0.001).

The relative importance of the different predictor variables was analyzed in a standardized model (Z-model). The standardized model shows that all of the structural characteristics had a greater impact on the number of bets made than did demographic characteristics.

Table 5 presents a cross-classified model between the number of bets made and bet characteristics. The null model is similar to the previous null model.

**Table 5:** A cross-classified multilevel model of the relationship between bet characteristics, demographic variables and the number of bets made

Parameters	Model 0	Model 1	Model 2	Z Model
*Fixed effects*				
Intercept	3.59 (0.43)	3.84 (0.32)	3.86 (0.32)	3.63 (0.30)
*Structural characteristics*				
Range bet	–	−0.06 (0.02)	−0.06 (0.02)	−1.00 (0.37)
Bet options	–	1.15 (0.74)^NS^	1.06 (0.72)^NS^	0.55 (0.37)^NS^
*Demographic characteristics*				
Female	–	–	0.08 (0.02)	0.03 (0.01)
Age	–	–	0.02 (0.00)	0.38 (0.01)
*Random effects*				
$\sigma_{0i}^{2}$	2.79 (0.02)	2.79 (0.02)	2.77 (0.02)	2.77 (0.02)
$\sigma_{0j(Game)}^{2}$	1.51 (0.75)^NS^	0.77 (0.39)^NS^	0.73 (0.37)^NS^	0.73 (0.37)^NS^
$\sigma_{0k(Individual)}^{2}$	0.54 (0.01)	0.54 (0.01)	0.43 (0.01)	0.43 (0.01)
*Model summary*				
−2LL	306,595.26	306,589.93	304,034.73	304,034.73
AIC	306,603.26	306,601.93	304,050.73	304,050.73
R^2^	–	15.1 %	18.8 %	
# of estimated parameters	4	6	8	8

Standard errors are listed in parentheses. Explanatory variables were centered in all of the models, except for the Z-model, in which the scores were standardized prior the analysis. Bets = log(bets)

*NS* Not significant

Model 1 shows that the difference in maximum and minimum bet size is negatively associated with the number of bets made, whereas the availability of advanced betting options was not significantly related to the number of bets made. Bet characteristics explained 15.1 % of the total variance in gambling behavior. The inclusion of betting options reduced the between game variance by 49.0 % and had a minimal effect on the other variance components, showing that betting options constitute a real contextual factor. However, the inclusion of betting variables did not improve the fit of the model compared with the null model (*χ* ^2^ = 5.33, *df* = 2, *p* = 0.07). This finding was supported by the small decrease in Akaikes information criterion (AIC).

Model 2 shows that both demographic and structural game characteristics, except for advanced betting options, were related to betting behavior. The final model predicts that a smaller difference between maximum and minimum bet size offered by a game (but not advanced betting options), being female and age are positively associated with the number of bets made. The final model explains 18.8 % of the variance in the number of bets made. The inclusion of demographic characteristics reduce both the between game and between individual variance by 5.2 and 20.4 %, respectively. The inclusion of demographic characteristics significantly improved model (*χ* ^2^ = 2,555.2, *df* = 4, *p* < 0.001).

The relative importance of the different predictor variables was analyzed in a standardized model (Z-model). The standardized model shows that the betting options have a greater impact on the number of bets made than demographic characteristics.

## Discussion

The aim of the study was to examine the relationship between structural game characteristics and gambling behavior in an ecologically valid setting. Behavioral tracking data was analyzed in a cross-classified multilevel model in which the number of bets made was nested within both games and individuals. The results show that the structural game characteristics (e.g., reward characteristics and betting options) as well as the demographic characteristics of the gambler affect the number of bets made. Furthermore, the analysis shows that structural characteristics have a greater impact on the number of bets made than age and gender. Overall, the findings show that making more bets is associated with higher payback percentage, less frequent wins and with a smaller average size of win. Advanced betting options are not associated with the number of bets made, whereas fewer betting options and lower maximum bet size are associated with higher number of bets made.

In line with the behavioral paradigm, the results show that games with a higher payback percentage is positively related to the relative number of bets made. Therefore, by minimizing the average expected loss per bet may increase the frequency of bets made. This result suggests that individuals prefer games with less expected losses. These results are also consistent with previous laboratory findings. Coates and Blaszczynski (2012) found that participants who were able to discriminate and choose between games with different payback percentages developed a preference for games with a higher payback percentage. In contrast, Haw (2007) found a significant relationship between gambling machine choice and payback percentage only among participants who switched gambling machines during a laboratory session. This might indicate that ‘switchers’ are motivated to maximize their outcome and might suggests differences in motivation among single game gamblers and gamblers that play on several games. Furthermore, this result may suggest that more bets should be made in games with a higher payback percentage regardless of whether the payback percentage is detectable or not (Coates and Blaszczynski 2012; Haw 2007). Future research should continue to study why gamblers prefer a higher payback percentage and examine, for example, do electronic machine gamblers prefer higher payback percentage because they are motivated to maximize the time on the device or maximize their outcomes. Further, this result suggests that the motivation to gamble should be examined between different groups of gamblers.

The average win size was negatively related to the number of bets made. In line with Delfabbro and Winefield (1999), this result suggests that a smaller sized win may maintain gambling behaviors whereas larger wins may disrupt it. This result may also suggest that gamblers are motivated to re-gamble small wins. However, it should be noted that Delfabbro and Winefield (1999) examined in-session gambling behavior, whereas the current study examined the mean size of a win within a month.

An interesting finding was that hit frequency was positively related to the number of bets made. Inconsistent with previous findings and theoretical assumptions that suggest gamblers prefer frequent wins (Coates and Blaszczynski 2012; Dixon et al. 2006) the present findings show that *less* frequent wins are associated with the number of bets made. In line with our descriptive analysis, the most played games had a lower average hit frequency. This result might suggest that differences in the average hit frequency between games is too small to be detected by the players (Coates and Blaszczynski 2012; Haw 2007) or that the motivation to gamble is associated with other factors, such as maximizing the outcome (Haw 2007). Furthermore, the presence of other game features, such as multiple wins per bet and bonus features may interfere with the perception of wins in a game (Coates and Blaszczynski 2012). Another explanation might be related to the way the variable was operationalized (i.e., average number of bets per win of any size). Games such as *Jokerdryss Bling Bling* and *Ballpower* included the opportunity to win multiple wins times per bet and that the number of wins might actually have been lower within these games than the suggested estimate. Interestingly, this result might also suggest that players are not responsive to the number of wins per bet but to the absolute number of wins. Maybe gamblers are more responsive to the number of wins in a given time frame, motivated to maximize their monetary outcome or time on device than the hit frequency of a game. Future research should examine these issues further, particularly how other game features, such as multiple wins per bet, influence game play.

In line with the findings of Livingstone and Woolley (2008), the descriptive analysis shows that the average bet size in all of the games was higher than the minimum denomination of the game. In line with behavioral theory, this finding suggests that the majority of gamblers are motivated to multiply their outcomes and/or increase the odds of winning a bet. For example, by maximizing the number of pay lines, the gambler also maximizes the odds of receiving a reward (Haw 2009). However, the findings do not support the suggestion that the presence of advanced betting options influences the number of bets made. This result might be due to the way the variable was operationalized. Because this variable was dummy coded, it may not have been an adequate conceptualization of the betting options.

In addition, the findings show that the absolute difference between the maximum and minimum allowed bet size of a game influence the number of bets made. The analysis shows that a greater difference between the maximum and minimum bet range is negatively associated to the number of bets made. It should be noted that a greater difference in bet size are also associated with a higher maximum bet size. Consistent with the descriptive analysis, a smaller difference between the maximum and minimum bet sizes is associated with more bets made. One could argue that gamblers prefer games with a lower maximum bet size, which may possibly be associated with the monetary value of the bet. Among others, Weatherly and Brandt (2004) found that the credit value of a bet was negatively associated with the number of bets made, and that individuals were sensitive to the monetary value of a bet. Hence, these findings predict that more bets would be made in games with a smaller difference in maximum and minimum bet size, possibly due to a lower maximum bet size. However, it should be noted that game preference could be attributed to other characteristics, such as the design and type of game. Hence, more experimental studies should be conducted to examine whether varying the difference between the maximum and minimum bet size in a game influences gambling behavior and preferences.

### Implications for Responsible Gambling Policy and Strategies

Responsible gambling strategies aim to give the gambler an opportunity to make informal decisions about an acceptable rate and size of loss within a specific time frame in order to reduce the potential harm of gambling (Blaszczynski et al. 2011; Blaszczynski et al. 2004. The results of our study may suggest that information, awareness and modifications to structural game characteristics may contribute to responsible gambling behavior.

It has been suggested that reducing the maximum bet size allowed in a game is an effective harm minimization strategy. Previous findings indicate that this strategy is associated with fewer bets made, less time spent gambling and less loss of money (Sharpe et al. 2005; Blaszczynski et al. 2001). However, our current findings show that more bets are placed in games with a lower maximum bet size and/or a less variable bet range. In line with Weatherly and Brandt (2004), this result suggests that gamblers are sensitive to the size of a bet. Individuals may be motivated to play games that maximize the opportunity to win for a low cost, extend their time on the device or to limit the rate of loss within a given timeframe. However, a small maximum bet size also increases gambling participation, which might lead to sustained gambling behaviors and habitual play. As such, more research should be conducted to examine how bet sizes influence gambling behavior in different groups.

The reward characteristics appear to influence the number of bets made. The current findings show that a higher payback percentage is associated with more bets made, which suggests that payback percentage influences the time spent gambling and may develop and sustain gambling behaviors. However, monetary losses can also be reduced by increasing the payback percentage of a game (Weatherly and Brandt 2004).

Inconsistent with previous findings and theoretical assumptions, more bets were made in games with *less* frequent wins per bet. This result might suggest that the combination of other gambling features in a game (e.g., multiple wins and the availability of bonus games) influences the number of bets made. However, little is known regarding how combinations of game features affect overall gambling behavior and cognitions, suggesting the need for future research.

The results show that the size of a jackpot is not associated with more bets made. This result suggests that modifying jackpot values between 125 NOK ($20 US) and 1,500 NOK ($250 US) would not be an effective strategy for reducing the number of bets made in a game.

However, because our results show that several structural game characteristics influence gambling behavior, future research should examine how these characteristics influence gambling behavior in combination. Examining the interactions between multiple game characteristics could lead to more precise and accurate gambling models and to more effective responsible gambling strategies. This should further be validated with experimental research.

## Limitations

Because only eight games were included, the validity and generalizability of the findings may be limited. Furthermore, as is common in multivariate analyses, extreme cases may have a substantial influence on the results. However, several predictions appear consistent with previous findings that support our model. Still, future studies involving more games should be undertaken to validate the findings of the present study.

Although the true prevalence of misuse, such as card swapping and theft, is not known, *Norsk-Tipping* blocked approximately 250 player cards in 2013 (Norsk-Tipping, personal communication, April 29, 2014). The majority of misuse (approximately 70 %) was related to card swapping within familiar circles. Furthermore, it should be noted that misuse has a limited purpose. The smartcards are protected with a four digit personal identification number (PIN). Wins and credits are electronically linked and transferred to a personal bank account and transactions to and from this account must be conducted online or by a *Norsk*-*Tipping* commissioner. Overall, this suggests that the misuse of gambling cards is a minor threat to the validity of this study.

It should be noted that the sample is only representative of gamblers who used *Multix* machines. Gamblers also use other gambling products and websites. However, because *Multix* was the only legal gambling machine during the study and the recorded data were collected unobtrusively and represent actual gambling behaviors, these limitations appear to be a minor threat to the generalization to EGM gamblers.

## Conclusion

Previous research has tended to be conducted in laboratory settings and focused on the relationship between in-session gambling behavior and the structural game characteristics. The results of these studies support the hypothesis that the structural characteristics of a gambling machine may influence gambling behavior. The current study has further expanded this assumption by examining the relationship between the population-level gambling behavior and the aggregate characteristics of different games in an ecologically valid setting. The findings suggest that the reward and betting characteristics of a game may influence game-level gambling behavior. These findings may therefore have implications for the design of gambling machines and responsible gambling policies. Future research should also expand and examine the relationship between structural characteristics and gambling behavior from different theoretical perspectives (e.g., prospect theory and self-justification) and how such moderators might influence gambling behavior.
